# A cross‐sectional study comparing a blood test for methylated *BCAT1 and IKZF1* tumor‐derived DNA with CEA for detection of recurrent colorectal cancer

**DOI:** 10.1002/cam4.868

**Published:** 2016-10-11

**Authors:** Graeme P. Young, Susanne K. Pedersen, Scott Mansfield, David H. Murray, Rohan T. Baker, Philippa Rabbitt, Susan Byrne, Libby Bambacas, Paul Hollington, Erin L. Symonds

**Affiliations:** ^1^ Flinders Centre for Innovation in CancerFlinders University of South AustraliaBedford ParkSouth AustraliaAustralia; ^2^ Clinical Genomics Pty LtdNorth RydeNew South WalesAustralia; ^3^ Colorectal SurgeryDivision of Surgery & Perioperative MedicineFlinders Medical CentreBedford ParkSouth AustraliaAustralia; ^4^ Bowel Health ServiceRepatriation General HospitalDaw ParkSouth AustraliaAustralia

**Keywords:** *BCAT1*, carcinoembryonic antigen, Colorectal cancer recurrence, *IKZF1*, monitoring, circulating tumor‐derived DNA

## Abstract

Recurrence will develop in 30–50% of colorectal cancer (CRC) cases despite apparent clearance following treatment. Carcinoembryonic antigen (CEA) is the only guideline‐recommended blood test for monitoring cases for recurrence, but its sensitivity and specificity are suboptimal. This observational study compared a novel 2‐gene (methylated *BCAT1* and *IKZF1* DNA) blood test with CEA for detection of recurrent CRC. We conducted a paired comparison of the *BCAT1*/*IKZF1* test with CEA (cut‐off 5 ng/mL) in blood from patients in remission after treatment for primary CRC and undergoing surveillance. Blood collected in the 12 months prior to or 3 months after complete investigational assessment of recurrence status were assayed and the results compared by McNemar's test. Of 397 patients enrolled, 220 underwent satisfactory assessment for recurrence and 122 had blood testing performed within the prescribed period. In 28 cases with recurrent CRC, CEA was positive in 9 (32%; 95% CI 16–52%) compared to 19 (68%; 95% CI 48–84%) positive for methylated *BCAT1/IKZF1* (*P* = 0.002). All samples that were CEA positive were also *BCAT1/IKZF1* positive. In 94 patients without clinically detectable recurrence, CEA was positive in 6 (6%, 95% CI 2–13%) and *BCAT1/IKZF1* in 12 (13%, 95% CI 7–21%), *P* = 0.210. The odds ratio of a positive CEA test for recurrence was 6.9 (95% CI 2–22) compared to 14.4 (5–39) for *BCAT1/IKZF1*. The *BCAT1/IKZF1* test was more sensitive for recurrence than CEA and the odds of recurrence given a positive test was twice that of CEA. The *BCAT1/IKZF1* test should be further considered for monitoring cases for recurrence.

## Introduction

Colorectal cancer (CRC) is the second leading cause of death from cancer in the developed world 1. Approximately 30–50% of patients will suffer recurrence despite achieving remission with initial treatment 2. Consequently, patients are entered into a follow‐up regimen with the goal of detecting recurrence at a stage where further curative‐intent therapy might be beneficial. Recent reviews of randomized controlled trials determined that an intensive surveillance regimen is more effective than minimal surveillance 2, 3. Patients with asymptomatic recurrence are more likely to be eligible for curative resection, to have an increased chance for successful surgery, and to have significantly better progression‐free and overall survival rates after such surgery 3, 4, 5, 6.

Regular blood testing for carcinoembryonic antigen (CEA, every 3‐ to 6 months) is currently the only blood test recommended for routine monitoring of CRC 7, 8, 9, 10, 11, 12, 13. While CEA measurement is the most sensitive simple test to aid in the monitoring of CRC 10, 14, 15, 16, 17, its sensitivity depends on what blood level, or changes in it, are chosen for positivity. Some have considered it too insensitive to be used alone 16.

We have previously reported several genes with hypermethylated regions differentiating adenoma and adenocarcinoma tissues from normal colorectal epithelium and benign pathologies 18, 19. Two of these genes, branched‐chain amino acid transaminase 1 (*BCAT1*) and ikaros family zinc finger protein 1 (*IKZF1*) may play an important functional role in maintaining a healthy colorectal tissue 20, 21, and both *BCAT1* and *IKZF1* appear to be involved in tumor growth and invasiveness 21, 22, 23, 24, 25, 26. Solid tumors, including CRC 25, 27, release DNA into circulation, and we have shown that cell free circulating DNA in blood from CRC patients has a significantly higher fraction of methylated *BCAT1* and *IKZF1* compared to healthy controls 28, 29.

The accuracy of the methylated *BCAT1* and *IKZF1* blood test (hereinafter referred to as the *BCAT1*/*IKZF1* test) for detection of CRC has been assessed in two studies including nearly 3500 patients scheduled for colonoscopy. It was found to be 62–66% sensitive and 92–94% specific 29, 30. For CRC stages I‐IV, the true‐positivity rates were in the ranges of 38–41%, 69–76%, 59–73%, and 71–94%, respectively. In addition, 12 *BCAT1/IKZF1* test‐positive CRC cases with paired pre‐ and post‐surgery plasma showed reduction in methylation signal after surgery, with complete disappearance in 10 patients 29. Thus, the *BCAT1/IKZF1* blood test might have merit for surveillance for recurrence in cases with CRC.

The goal of this study, which incorporated clinic visits, blood sampling, and diagnostic imaging, was to compare the sensitivity and specificity of the *BCAT1*/*IKZF1* blood test to CEA, when applied on a single occasion, for detection of recurrent CRC in patients undergoing surveillance. True‐ and false‐positive rates for radiologically or histopathologically confirmed recurrence were compared and absolute sensitivity and specificity derived from these.

## Methods

### Study overview

This was an observational study that compared the accuracy of a blood test detecting methylated *BCAT1* and *IKZF1* DNA with a blood test measuring CEA levels in CRC patients under surveillance for disease recurrence. As there is no single gold standard for determination of recurrence, and pathological confirmation is not always obtained, a combination of analytical and clinical information for establishing recurrence status was applied (see below). The true‐ and false‐positive rates of the *BCAT1/IKZF1* and CEA blood tests were determined in the blood sample temporally closest to the investigation verifying recurrence status.

The study was approved by the Southern Adelaide Clinical Human Research Ethics Committee. Written informed consent was obtained from all volunteering participants prior to any procedures. The trial is registered at Australian and New Zealand Clinical Trials Registry, trial registration number 12611000318987.

### Population

Any adults (18 years of age or older) who were recently diagnosed but without residual macroscopic disease after initial treatment for CRC (AJCC stages I–IV) 31 or who were already undergoing surveillance monitoring for CRC recurrence at Flinders Medical Centre (Bedford Park, SA, Australia) were approached consecutively, wherever feasible, about volunteering for the study during the period of 24 months from November 2013. A CT examination of chest, abdomen, and pelvis was performed at 12 monthly intervals subject to the discretion of the clinician and depending on individual risk. Blood samples were obtained (usually at 3–6 month intervals) either in the 12 months prior to planned radiological imaging, at the time of confirmed recurrence, or within 3 months of confirmation but prior to any further treatment. Following enrollment, volunteers were excluded if initial treatment was not completed, if residual disease was evident, if radiological imaging was not obtained as part of assessment for recurrence, if imaging findings were indeterminate for recurrence, or if blood was collected during or within 3 months of any chemotherapy or radiotherapy treatment.

### Clinical procedures

Venous blood was collected into two 9 mL K3EDTA Vacuette tubes (Greiner Bio‐One, Frickenhausen, Germany) at clinic visits. Blood collection tubes were kept at 4°C prior to plasma processing (not more than 4 h from blood collection). Plasma was prepared by centrifugation at 1500*g* for 10 min at 4°C (deceleration at lowest setting), followed by retrieval of the plasma fraction and a repeat centrifugation. The resulting plasma was stored at −80°C. Frozen plasma samples were shipped on dry ice to Clinical Genomics Technologies (North Ryde, NSW, Australia) and stored at −80°C until testing. Cases were excluded from analysis if these conditions were not met. No study‐wide control of radiological imaging or pathology procedures or quality was undertaken as the study aimed to assess marker performance relative to outcomes determined in usual clinical practice. All procedures were performed by hospital‐accredited specialists and so met site‐specific standards for venipuncture, monitoring, imaging, and equipment.

### Classification of recurrence status

A combination of analytical and clinical information was used to establish the presence or absence of clinically detectable recurrence, that is, recurrence “status”, as there is no single gold standard technique for diagnosing recurrence status and histopathological confirmation is not always obtained. An independent physician, blinded to assay results, confirmed clinical recurrence status for all cases on basis of the findings of diagnostic tests (radiology, namely CT, MRI, or PET scan; or colonoscopy) supported by tissue diagnosis when available (not all cases with recurrence determined by imaging were subjected to histopathological confirmation). Recurrence was therefore confirmed to be present if any of the following applied: (1) Radiologically detected recurrence (CT, MRI, or PET scan, of abdomen, pelvis, and chest) confirmed by tissue pathology (date of diagnosis was the date of the radiology), (2) radiologically detected recurrence not subject to tissue pathology but affirmed as recurrence by the clinical management team (date of diagnosis was the date of the radiology), or (3) colonoscopically detected local recurrence at the anastomosis confirmed by tissue pathology (date of diagnosis was the date of the colonoscopy). Cases were excluded if any data crucial to determining clinical status were not obtainable, for example, inconclusive radiological imaging.

Evidence of recurrence in the perianastomotic site or rectal stump, presacral area, as well as regional nodal recurrence or lateral pelvic lymph node recurrence of rectal cancer was defined as local recurrence. Evidence of recurrence in the liver, lung, or other organs such as para‐aortic lymph nodes, peritoneum, bone, brain, adrenal gland was defined as distant recurrence. In cases diagnosed with both local and distant recurrences, distant recurrence was used as the principal diagnosis. Metachronous CRC (i.e., tumors detected in the colon at locations remote to the primary diagnosis) was not classified as recurrence and hence omitted from primary analysis.

Recurrence status was determined as “not clinically detectable” where the clinical management team considered that routine tests conducted as part of surveillance in the prior 12 months did not show evidence of recurrence. As a minimum, this must have included a satisfactory CT scan of chest, abdomen and pelvis, or alternative radiological imaging of these regions such as MRI or PET (collectively referred to hereafter as “radiological imaging”).

### DNA methylation testing

The presence of methylated *BCAT1* and *IKZF1* DNA in 3.9 mL plasma was determined as previously reported 30. A sample was deemed positive if at least one PCR replicate was positive for either *BCAT1* and/or *IKZF1* DNA methylation. Blood testing was performed by staff blinded to all clinical data.

### CEA testing

The concentration of CEA was determined using the LIAISON CEA test as recommended by manufacturer (DiaSorin S.p.A., Saluggia, Italy). A sample with CEA levels of 5 ng/mL or above was deemed positive, as commonly applied 16.

### Statistical analyses

A sample size of 24 pairs with recurrence would have 80% power to detect a difference in proportions of 0.300 based on an expected proportion of discordant pairs of 0.350 (using McNemar's test with a 0.050 two‐sided significance level), hence we undertook the analysis when we were confident that at least 25 cases with confirmed recurrence had been recruited. The principal outcome measure was positivity rate by recurrence status, verified as present or not clinically detectable by the clinical decision described above. Where more than one sample was collected, the sample temporally closest to verification of diagnostic status was considered. If an assay result was indeterminate, or missing, the case was excluded from analysis. Differences in paired positivity proportions and concordance analyses were analyzed using McNemar's test (two‐sided; significance level, 0.05). Binomial distribution was assumed for calculations of exact 95% confidence interval (95% CI). Test sensitivity estimates were expressed as the ratio of true positives over the sum of true positives plus false negatives. Specificity was estimated as 1 – positivity rate in cases with no evidence of recurrent disease. Stata version 13 was used for the statistical analyses described above. *P*‐values <0.05 were considered statistically significant.

## Results

### Study volunteers

**Figure 1:**
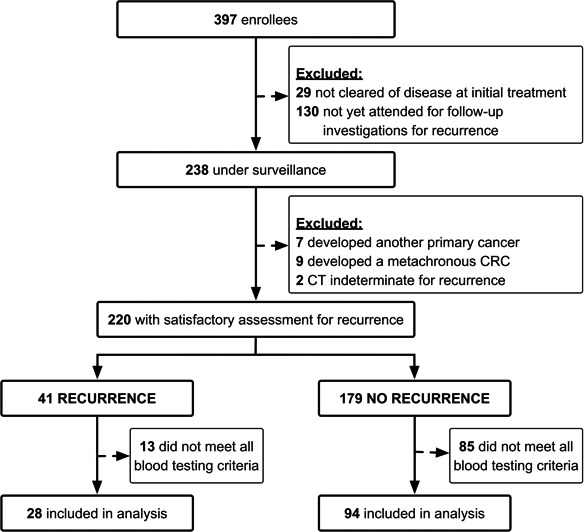
Disposition of study volunteers.

A total of 397 volunteers were recruited (61.2% men, median 66.1 years of age at diagnosis). Figure 1 shows the disposition of volunteers and the reasons for exclusion during surveillance and prior to determination of recurrence status; 33% had not completed relevant scheduled investigations for recurrence while 11% were either found not to be in remission or to have developed a cancer other than CRC. Recurrence status could be established in 220 patients under surveillance. Table S1 shows demographic features of the volunteers including the nature of recurrence, as well as CRC stage at diagnosis, with approximately equal numbers of patients diagnosed with early (I–II) and late (III–IV) stage CRC. The median ages of those with recurrence or without clinically detectable recurrence were similar (data not shown). The majority of recurrences were distant (35/41, 85.4%).

Suitable blood samples were available from 122 of the 220 patients with verified recurrence status (blood collected 12 months prior to, or 3 months after verification of status), as shown in Figure 1. Full details of these are shown in Table 1 where it can be seen that only 8 of the 28 with recurrence were of an early stage (all stage II) at diagnosis. Recurrence was most common with an initial staging of III or IV and in those with rectal cancer (Table 1). Morphological features associated with confirmed recurrence were the presence of lymphovascular and/or perineural invasion. The time elapsed between date of verification of recurrence status and the date of blood sampling did not differ between those with or without recurrence (Table 1).

**Table 1:** Characteristics of patients included in primary analysis

*N* = 122	Recurrence (*n* = 28)	No recurrence (*n* = 94)	*P* value
Age at diagnosis (years), median (IQR)^a^	66.0 (57.0–72.8)	65.1 (54.2–73.3)	0.684^b^
Gender, Male, No. (%)	17 (60.7)	59 (62.8)	0.841^c^
Characteristics of primary cancer			
Staging, No. (%)			
Stage I	0 (–)	28 (29.8)	0.001^c^
Stage II	8 (28.6)	32 (34.0)	0.589^c^
Stage III	17 (60.7)	30 (31.9)	0.006^c^
Stage IV	3 (10.7)	3 (3.2)	0.105^c^
Unstaged	0 (–)	1 (1.1)	0.582^c^
Location, No. (%)			
Right colon^d^	8 (28.6)	42 (44.7)	0.129^c^
Left colon	7 (25.0)	31 (33.0)	0.424^c^
Rectum	13 (46.4)	21 (22.3)	0.012^c^
Size, mm (median, IQR)^e^	50.0 (32.5–65.0)	41.0 (32–60)	0.213^b^
Lymphovascular, No. Present/Total (%)	10/12 (83.3)	18/85 (21.2)	0.000^c^
Perineural invasion, No. Present/Total (%)	6/22 (27.3)	5/81 (6.2)	0.005^c^
Differentiation, *N*/Total (%)			
Poor	6/22 (27.3)	14/89 (15.7)	0.208^c^
Moderate	15/22 (68.2)	66/89 (74.2)	0.569^c^
Well	1/22 (4.5)	9/89 (10.1)	0.412^c^
Treated with chemo/radiotherapy, No. (%)	23 (82.1)	34 (36.2)	0.000^c^
Months elapsed between diagnosis and verified recurrence status, median (IQR)	28.3 (21.9–41.0)	17.3 (12.0–29.2)	0.0004^b^
Location of recurrence, No. (%)			
Local	4 (13.8)	n/a	–
Distant	24 (85.7)	n/a	–
Months elapsed between proximate blood sample and verified recurrence status, median months (IQR)	1.8 (0.3–4.2)	1.6 (0.4–2.9)	0.305^b^
Serial blood tests within 12 months of verified recurrence status, No. (%)	7 (25.0)	23 (24.5)	0.952^c^

a Years; IQR, interquartile range.

b 2‐sided *t*‐test equal variances *P* value.

c Z‐score two population proportion test, 0.05 significance level.

d Cecum, ascending, hepatic flexure, transverse.

e Millimeter.

### Test sensitivity for recurrence

Results of the methylated *BCAT1/IKZF1* and CEA tests are shown in Table 2 for the 122 patients eligible for analysis.

**Table 2:** Performance of the methylated *BCAT1/IKZF1* and carcinoembryonic antigen (CEA) blood tests

	Positive counts relative to diagnostic verification of recurrence status, No (%, 95% CI)				
	*N*	*BCAT1/IKZF1*	OR (95% CI), *P* a	CEA^b^	OR (95% CI), *P* a
All eligible cases	122	31 (25.4, 18–34)	n/a	15 (12.3, 7–19)	n/a
Recurrence	28	19 (67.9, 48–84)	14.4 (5–39), <0.0001	9 (32.1, 16–52)	6.9 (2–22), 0.001
Local	4	3 (75.0, 19–99)	20.5 (2–213), 0.012	2 (50.0, 7–93)	14.7 (2–123), 0.013
Distant	24	16 (66.7, 45–84)	13.7 (5–39), <0.0001	7 (29.2, 13–51)	7.3 (2–25), 0.002
No recurrence	94	12 (12.8, 7–21)	1	6 (6.4, 2–13)	1

a Calculation of Odds Ratios (OR) against cases with no evidence of recurrence, Chi‐square *P* value <0.05.

b Cut‐off, 5 ng/mL.

Of the 28 patients with recurrent CRC, 67.9% (19/28, 95% CI 48–84%) and 32.1% (9/28, 95% CI 16–52%) were positive for methylated *BCAT1/IKZF1* DNA and CEA, respectively. This difference of 35.8% (95% CI 14–57%) was significant. Similar results were seen in cases with distant recurrence but numbers with local recurrence were small. The odds ratio for recurrence was 14.4 (95% CI 5.4–38.7) for the methylated *BCAT1/IKZF1* test (*P* < 0.001) and 6.9 (95% CI 2.3–21.1) for CEA (*P* = 0.001). Sensitivity of the methylated *BCAT1*/*IKZF1* blood test for recurrence was 75.0% in patients with stage II cancer at diagnosis (6/8), 70.6% of the stage III cancers (12/17), and 33.3% of the stage IV cancers (1/3). Sensitivity estimates of the methylated *BCAT1/IKZF1* test for local and distant recurrence were 75% and 66.7%, respectively, compared to 50% and 29.2% for CEA (Table 2).

Comparing those with or without verified recurrence, there was no significant difference in elapsed time between blood collection and clinical confirmation of recurrence status (Table S2).

### Estimates of specificity and predictive values

Of the 94 cases without clinically detectable recurrence, 12.8% (12/94, 95% CI 7–21%) and 6.4% (6/96, 95% CI 2–13%) were positive for methylated *BCAT1/IKZF1* DNA and CEA, respectively (Table 2). The difference of 6.4% (95% CI 3–16%) was not significant, *P* = 0.134). The positive predictive values of methylated *BCAT1/IKZF1* and CEA were 61.3% (42.2–78.2%) and 60.0% (32.3–83.7%), respectively. The negative predictive values were 90.1% (82.1–95.4%) and 82.2% (73.7–89.0%), respectively.

### Test concordance

Concordance between the two blood tests is shown in Table 3. Of the 28 cases with recurrence, 9 (32.1%) were positive in both tests, whereas the methylated *BCAT1/IKZF1* test detected an additional 10 cases that were CEA negative, indicating a significantly better sensitivity with the methylated *BCAT1/IKZF1* test (*P* = 0.002). There were no cases that were CEA positive only.

**Table 3:** Concordance between tests

		CEA^a^		*P* b
		No. positive	No. negative	
	***BCAT1/IKZF1***			
Recurrence (*n* = 28)	No. positive	9	10	0.002
	No. negative	0	9	
No recurrence (*n* = 94)	No. positive	1	11	0.210
	No. negative	5	77	

a Cut‐off, 5 ng/mL.

b McNemar's test *P*‐value two‐sided.

In those with no clinically detectable recurrence, only one was positive for both tests. Eleven were positive by methylated *BCAT1/IKZF1* only*,* and 5 by CEA only (*P* = 0.210).

### Longitudinal test results over time

Of the 122 patients with recurrence status defined, 30 cases provided more than one blood sample taken within the qualifying window (12 months prior, 3 months after); 7 had recurrence and 23 cases had no clinically detectable recurrence.

**Figure 2:**
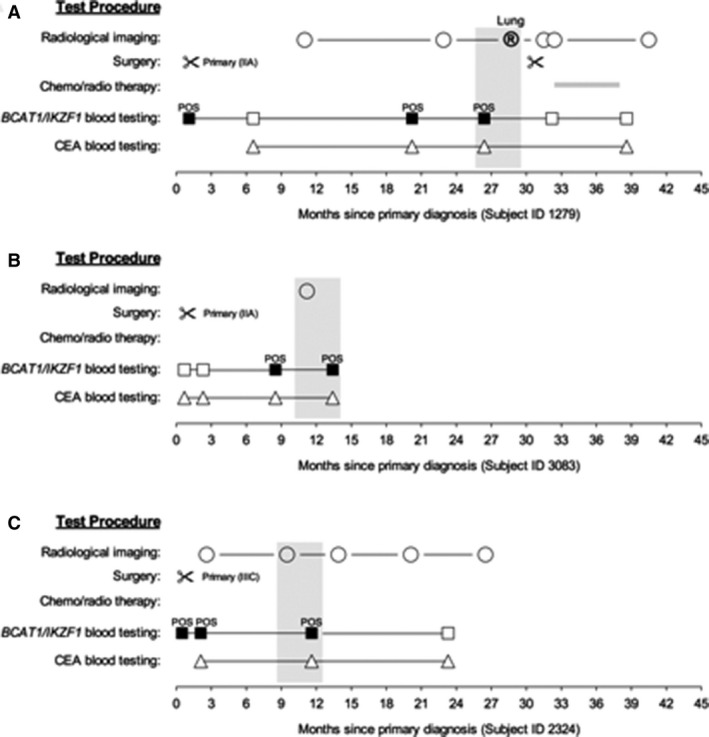
Longitudinal monitoring profiles of cases providing serial methylated *BCAT1/IKZF1* blood tests. (A) A case where an apparent false positive became a true positive. (B) A case where a false positive was confirmed by a second blood test. (C) A case where a false positive was not confirmed by a second blood sample. Circle, radiological imaging; circled R, confirmation of recurrence; squares, *BCAT1/IKZF1* blood testing; triangles, carcinoembryonic antigen testing. Open symbols, negative blood result; filled symbols, positive blood result. Grey horizontal bar: period of receiving chemo/radiological therapy. Grey vertical box: most proximal blood test—radiological imaging results included in primary analysis.

In 28 of those 30 cases, the second closest *BCAT1/IKZF1* test result was in concordance with the result closest to diagnostic verification of recurrence status (which was used for the main analysis). This included seven recurrence cases, one of whom had an intervening negative CT scan (Fig. 2A), and 21 cases with no clinically detectable recurrence including three cases with apparent false‐positive results that remained positive with a second later blood test (example provided in Fig. 2B).

For the remaining two cases who had no clinical‐detectable recurrence, the second blood tests were negative (example provided in Fig. 2C).

### Variables influencing positivity

Of the seven patients diagnosed with a cancer other than CRC, six had blood sample collection. Cancers diagnosed included breast cancer with metastasis to the bones, thyroid cancer, metastasis to the abdominal cavity from a large cell carcinoma, metastasis to the lymph nodes from ovarian cancer, and two patients with renal cell carcinoma. The methylated *BCAT1/IKZF1* test was positive in the former three cases, with CEA levels above 5 ng/mL in two patients (breast cancer and thyroid cancer).

The methylated *BCAT1/IKZF1* test positivity was not affected by gender or age (Table S3).

## Discussion

This test for methylated *BCAT1* and *IKZF1* DNA in blood facilitates detection of recurrent CRC. A direct paired comparison to the CEA blood test demonstrated that the *BCAT1/IKZF1* test had a significantly better sensitivity for recurrence, whereas the two tests did not significantly differ in specificity. Using the odds ratio as a single indicator of diagnostic performance 32, the odds of recurrence being present with a positive *BCAT1/IKZF1* test was twice that with CEA.

Based on the observed true‐positive rate of the *BCAT1/IKZF1* blood test using the blood sample taken closest to radiological confirmation of recurrence status, sensitivity estimates for local and distant recurrence were 75% and 66.7%, respectively. The estimated sensitivity for any recurrence was 67.9%. These estimates justify considering the use of the *BCAT1/IKZF1* blood test in surveillance for recurrent CRC. A minority of stage II CRC cases received adjuvant therapy and had a lower risk of recurrence. Nonetheless, the *BCAT1/IKZF1* blood test was positive in 6/8 (75%) of the recurrences occurring in patients initially diagnosed with stage II CRC. This suggests that the *BCAT1/IKZF1* blood test might be usefully applied in surveillance of all cases in remission, regardless of initial stage.

We compared the *BCAT1/IKZF1* blood test with the CEA blood test because the latter is the only noninvasive blood test recommended by several authorities in their guidelines for CRC surveillance 7, 8, 9, 11, 12, 33. Using the commonly applied cut‐off value of 5 ng/mL 16, we estimated sensitivity of CEA for recurrence to be 32.1% overall, with estimates for local and distant recurrence of 50 and 29.2%, respectively. These values tend to be lower than what is reported in the literature 16 and the reasons for this are unclear. The lower sensitivity for CEA may be partly due to this study being cross‐sectional, that is, consideration of the result in a plasma sample taken at just one point in time rather than a change in test result over time.

When comparing true‐positive rates in the same patient, the methylated *BCAT1/IKZF1* blood test had a significantly higher sensitivity for recurrent CRC than the CEA test (Tables 2 and 3), and in this study, the *BCAT1/IKZF1* blood test correctly identified 10 additional cases of recurrent CRC. The design of this study did not allow us to systematically determine if the *BCAT1/IKZF1* blood test was able to detect cases with recurrent CRC earlier than the CEA test. A prospective study with multiple sampling commencing at the end of initial CRC treatment would be needed to address this question and is justified based on our findings. Earlier detection is considered important to survival 5.

Based on the observed false‐positive rates for the methylated *BCAT1/IKZF1* and CEA blood tests, using radiological imaging as an essential element for deciding upon recurrence status, the specificity estimates were 87.2% and 93.6%, respectively, and were not significantly different (*P* = 0.210).

A limitation of the study is that the calculations of the true‐ and false‐positivity rates were dependent on the findings at clinical investigation relative to the result obtained with the temporally closest blood test. It is possible that the specificity estimate for either test reported herein is too low and that some false‐positives are apparent rather than real due to the presence of subclinical recurrence not yet detectable by imaging. Radiological imaging can only detect recurrences within the limit of detection. As a consequence, a result designated now as a false‐positive might ultimately be true. In Figure 2A, we show a case which returned an apparent false positive that became a true positive on a subsequent imaging scan. Longitudinal follow‐up studies with serial blood testing are required to understand whether false‐positives correlate with recurrence detectable only at a subsequent radiological follow up.

In addition to the issue of length of follow‐up, this study has some other limitations. It is an observational study conducted in a usual‐care moderate‐sized clinical service where follow‐up protocols are subject to variance, rather than in the context of a formal highly structured prospective clinical trial. As such, the timing of blood tests relative to diagnostic imaging varied. Most samples were collected before diagnostic assessment but some were collected in the window afterwards, such that the tests were being compared at different stages of the biological progression to clinically detectable recurrence. We chose the window of 12 months prior to radiological assessment for recurrence (as this is the usual interval aimed for in practice) and 3 months after such (as long as treatment was not instigated) for the purposes of comparing the two tests, but in future, multiple blood sampling over time with repeated diagnostic imaging as per surveillance protocols will serve to further clarify the actual and relative value of each test.

Finally, this study does not explore impact on survival. In‐so‐far as a modest survival benefit with CEA testing as part of a surveillance protocol has been observed 3, and that the *BCAT1/IKZF1* test is more sensitive for recurrence in our study, then definitive studies aimed at assessing potential survival benefit with the *BCAT1/IKZF1* test are now indicated. There might also be potential for prediction of prognosis independent of stage, as has been reported for other biomarkers 34.

The presence of methylated *BCAT1* and *IKZF1* DNA in blood is not likely to be limited to CRC only 24, 35, and as reported here, three of six cases diagnosed with other cancers were positive with the *BCAT1/IKZF1* test. Studies to examine its relevance to other cancers are now underway.

The methylated *BCAT1/IKZF1* test is twofold more sensitive than the CEA test for CRC recurrence, whereas there is no significant difference in the specificity estimates between the two tests. Furthermore, the odds of recurrence given a positive methylated *BCAT1/IKZF1* test is 14.4 compared to 6.9 with a positive CEA test. If used in surveillance as a trigger to bring forward scheduled radiological imaging, recurrence seems likely to be detected earlier without undue load on radiological services. Consequently, it is now justifiable to proceed to a prospective longitudinal evaluation of the methylated *BCAT1/IKZF1* blood test versus CEA to ascertain the temporal relationships between positivity and recurrence (and hence the ideal frequency of testing), relative sensitivity and specificity on the basis of longer follow‐up and whether better and/or earlier detection leads to any survival benefit.

## Conflict of Interest

GPY is a paid consultant to Clinical Genomics. SKP, DHM, and RTB are paid employees of Clinical Genomics.

## Supporting information


**Table S1.** Demographic characteristics of study cohort.
**Table S2.** Test positivity rates relative to time elapsed between verified recurrence status and time of taking blood sample.
**Table S3.** Methylated *BCAT1/IKZF1* blood test positivity versus gender and age.
