# Supplementary material for: A cross‐sectional study comparing a blood test for methylated BCAT1 and IKZF1 tumor‐derived DNA with CEA for detection of recurrent colorectal cancer
Source: Cancer Med. 2016 Oct 11;5(10):2763–72. doi: 10.1002/cam4.868 (PMC5083729; doi:10.1002/cam4.868)
Supplement: Supplementary file 1 — Table S1. Demographic characteristics of study cohort. Table S2. Test positivity rates relative to time elapsed between verified recurrence status and time of taking blood sample. Table S3. Methylated BCAT1/IKZF1 blood test positivity versus gender and age. [file CAM4-5-2763-s001.docx]

**Supplementary On-Line Table 1: Demographic characteristics of study cohort.**

|  | | |  | | **Age^1^** | **Women** | **Men** |
| --- | --- | --- | --- | --- | --- | --- | --- |
|  | | | n (%) | | Median (min-max) | n (%), Median age | |
| **Staging of primary cancer:** | | | | |  |  |  |
| Cancer – any stage | | | | 397 | 66.1 (31.3-85.9) | 154 (38.8), 66.5 | 243 (61.2), 66.0 |
|  | | I | | 74 (18.6) | 65.5 (40.3-84.9) | 33 (44.6), 65.5 | 41 (59.5), 66.0 |
| II | | | | 113 (28.5) | 66.7 (31.5-85.3) | 47 (41.6), 66.7 | 66 (58.4), 66.8 |
| III | | | | 129 (32.5) | 67.4 (31.3-85.9) | 51 (39.5), 64.1 | 78 (60.5), 68.6 |
| IV | | | | 38 (9.6) | 65.7 (41.3-84.4) | 10 (26.3), 67.7 | 28 (73.7), 63.7 |
| Unstaged | | | | 43 (10.8) | 62.6 (32.9-85.3) | 13 (30.2), 68.2 | 30 (69.8), 59.8 |
|  | | | |  |  |  |  |
| **Clinical status at study completion:** | | | | |  |  |  |
| Not cleared of disease at initial treatment | | | | 29 (7.3) | 66.4 (31.3-84.4) | 9 (31.0), 67.4 | 20 (69.0), 65.2 |
| Developed another primary cancer | | | | 7 (1.8) | 60.3 (48.1-78.1) | 2 (28.6), 53.8 | 5 (71.4), 68.6 |
| Developed a metachronous CRC | | | | 9 (2.3) | 70.2 (58.7-85.3) | 7 (77.8), 70.8 | 2 (22.2), 62.4 |
| No radiological imaging or indeterminate | | | | 132 (33.2) | 68.2 (31.5-84.9) | 54 (40.9), 68.2 | 78 (59.1), 68.3 |
|  | | | |  |  |  |  |
| **Cases with recurrence** | | | | **41 (10.3)** | **66.0 (45.2-84.1)** | **13 (31.7), 66.7** | **28 (68.3), 64.4** |
| Local | | | | 6 (14.6) | 59.4 (45.2-73.9) | 2 (33.3), 58.4 | 4 (66.6), 59.4 |
|  | Distant | | | 35 (85.4) | 66.0 (45.2-84.1) | 11 (31.4), 66.7 | 24 (68.6), 64.4 |
| Study cases eligible for analysis^2^ | | | | 28 (68.2) | 66.0 (45.2-84.1) | 11 (39.3), 66.8 | 17 (60.7), 62.9 |
|  | | | |  |  |  |  |
| **Cases with no recurrence** | | | | **179 (45.1)** | **64.5 (31.8-85.9)** | **69 (38.5), 63.3** | **110 (61.5), 65.7** |
| Study controls eligible for analysis^2^ | | | | 94 (46.9) | 65.1 (31.8-85.1) | 35 (37.2), 61.6 | 59 (62.8), 66.2 |
|  | | | |  |  |  |  |

^1^ Age at the time of primary diagnosis (years);

^2^ Blood testing performed in accordance with acceptance criteria.

**Supplementary On-Line Table 2: Test positivity rates relative to time elapsed between verified recurrence status and time of taking blood sample.**

|  | Recurrence | | No Recurrence | |
| --- | --- | --- | --- | --- |
|  | *BCAT1/IKZF1* blood test | CEA | *BCAT1/IKZF1* blood test | CEA |
| 3 months prior to 3 month after | 14/19 (73.7)^1^ | 6/19 (31.6) | 10/72 (13.9) | 6/72 (8.3) |
| 6 to 3 months prior | 3/5 (60.0) | 2/5 (40.0) | 1/14 (7.1) | 0/14 (0) |
| 9 to 6 months prior | 1/2 (50.0) | 1/2 (50.0) | 0/4 (0) | 0/4 (0) |
| 12 to 9 months prior | 1/2 (50.0) | 0/2 (0) | 1/4 (25.0) | 0/4 (0) |
| P-value^2^ | 0.342 | 1.00 | 0.560 | - |
|  |  | |  |  |

**^1^** Number Positive/Total (%)

**^2^** Logistic multivariate analysis

**Supplementary On-Line Table 3: Methylated *BCAT1/IKZF1* blood test positivity versus gender and age**

|  | **Clinical status of recurrence**  No. Positives/total (%) | |
| --- | --- | --- |
|  | Recurrence (n=28) | No recurrence (n=94) |
|  |  |  |
| Gender: |  |  |
| Females | 7/11 (63.6) | 5/35 (14.3) |
| Males | 12/17 (70.6) | 7/59 (11.9) |
| P-value^1^ | 0.703 | 0.728 |
| Age at the time of blood draw: |  |  |
| <65 years | 10/13 (76.9) | 7/47 (14.9) |
| ≥65 years | 9/15 (60.0) | 5/47 (10.6) |
| P-value^1^ | 0.337 | 0.535 |
|  |  |  |
| ^1^ 2-sample z-test on sample proportions (2-tailed, 0.05 significance level). | | |
